# Advancing Parkinson’s detection from MRI: a deep learning comparison of classical and quantum architectures

**DOI:** 10.1186/s12880-026-02639-y

**Published:** 2026-08-13

**Authors:** Jothiraj Selvaraj, Fadiyah Almutairi, Omar Alhajlah, Snekhalatha Umapathy

**Affiliations:** 1 Department of Biomedical Engineering, School of Engineering and Technology, Karunya Institute of Technology and Sciences, Karunya Nagar, Coimbatore, Tamil Nadu 641114 India; 2 School of Computer Science and Artificial Intelligence, SR University, Ananthasagar, Hasanparthy, Warangal, Telangana 506371 India; 3 Department of Information Systems, College of Computer and Information Sciences, Majmaah University, Al Majmaah, 11952 Saudi Arabia; 4 Department of Applied Computer Sciences, Applied Computer Science College, King Saud University, Riyadh, 11451 Saudi Arabia; 5 Department of Biomedical Engineering, College of Engineering and Technology, SRM Institute of Science and Technology, Kattankulathur, Chengalpattu, Tamil Nadu 603203 India; 6 College of Engineering, Architecture and Fine Arts, Batangas State University, Batangas, Philippines

**Keywords:** Parkinson’s disease, MRI image classification, CNN, Vision transformer, Swin transformer, QNN

## Abstract

**Purpose:**

The precise diagnosis of Parkinson’s Disease (PD) continues to pose a considerable barrier in clinical neurology. This work investigates the capability of sophisticated deep learning architectures to improve Parkinson’s disease identification via Magnetic Resonance Imaging (MRI) scans, aiming to improve diagnostic sensitivity and specificity.

**Method:**

This research implements an automated PD classification framework utilizing deep learning models to analyze real-time MRI scans. CNN models and Transformers that leverages the inherent spatial information within MRI scans for automated feature extraction and classification of PD was implemented. The comparative analysis of diverse architectures aimed to discern the optimal feature extraction and classification strategies for distinguishing PD-affected brains from healthy controls was performed. K-fold cross-validation was conducted to evaluate the reliability of dataset partitioning and to ensure model generalizability.

**Results:**

The QNN demonstrated superior performance during training and validation with accuracy of 0.9851 and 0.9731 respectively. Among the Transformer-based architectures, the Swin Transformer achieved a training and validation accuracy of 0.9712 & 0.9673. In the category of CNN-based models, ResNet50 exhibited the highest accuracy, attaining 0.9322 during training and 0.9192 during validation. The K-fold cross-validation results corroborated the stability and consistency of the model performances with the 80:20 data split, reinforcing the robustness of the proposed approach. Furthermore, to assess the generalizability of the best-performing model, an external validation was conducted using the Parkinson’s Progression Markers Initiative (PPMI) dataset. The results demonstrated comparable performance trends, thereby confirming the adaptability of the DL model to independent datasets.

**Conclusion:**

This study’s findings highlight the capability of deep learning-driven neuroimaging analysis in facilitating automated and precise PD diagnosis. This investigation provides a scalable and clinically viable AI framework that enhances diagnostic accuracy, thereby supporting clinicians in disease detection and improving patient management strategies.

## Introduction

Parkinson’s disease (PD) is an irreversible neurological ailment that impairs mobility, mostly due to the degeneration of neurons that produce dopamine in the brain [1, 2]. PD is typically associated with symptoms like tremors, muscle rigidity, bradykinesia, and postural instability [3, 4]. PD impacts one million individuals in the United States, a number anticipated to rise to 1.2 million by 2030. Globally, more than 10 million individuals are afflicted with PD, rendering it the second most prevalent neurological condition subsequent to Alzheimer’s disease [5, 6]. The diagnosis of PD is essential for prompt intervention and therapy, as delayed identification may result in significant motor and cognitive impairments [7, 8]. The traditional diagnostic methods involving history-taking and neurological assessments are subjective and prone to misinterpretation of the disease [9, 10].

Radiological imaging has emerged as a paramount clinical assessment in the diagnosis of wide spectrum of neurological disorders [11–13]. Magnetic Resonance Imaging (MRI) provides insights into brain abnormalities with detailed information about the intricate architecture of the brain, enabling the identification and characterization of PD [14, 15]. Despite its effectiveness, manual analysis of MRI scans requires specialized expertise and is often time-consuming, leading to inconsistencies in diagnosis [16]. This highlights the need for automated and objective methods to improve the precision and efficacy of PD detection [17, 18].

The AI-driven methodologies encompassing Machine Learning (ML) and Deep Learning (DL), have gained significant attention for automating the accurate PD diagnosis from MRI images [19]. The ML models are limited as its performance depends on the domain expertise in identifying and engineering pertinent features capturing the neuropathological signatures that are indicative of PD from the MRI images [20]. DL methods including hybrid architectures and convolutional neural networks (CNNs) have demonstrated incredible potential within the realm of digital medical imaging [21]. In particular the CNNs can autonomously extract intricate information from MRI scans, identifying subtle structural alterations [22]. The DL model excels the ML model in terms of automated feature extraction, data requirements, and computational complexity [23]. This research article provides a comparative analysis of Quanvolutional Neural network (QNN) and DL techniques in PD identification, highlighting their strengths, limitations, and applications. The integration of DL in PD diagnosis holds promise for developing a reliable and efficient approach in the identification of disease.

### Literature survey

The PD has been detected from EMG, speech signal other than MRI images. Deepak Joshi et al. combined wavelet method of feature extraction with ML algorithm and achieved accuracy of 90.32%. In recent works, CNNs have been widely adopted due to their ability to learn hierarchical features from images, making them particularly effective for tasks such as image classification and segmentation [24]. A study by Sangeetha et al., 2023 demonstrated that CNNs could effectively classify MRI images of PD patients, achieving high accuracy rates of 97% using the Parkinson Progression Markers Initiative (PPMI) dataset [25]. Recent studies have also explored the use of Transformer architectures, which have demonstrated superior performance in various image classification tasks by leveraging self-attention mechanisms [26]. Despite the advantages of CNNs, there are arguments in favor of using transformer architectures for the diagnosis of Parkinson’s disease. Vision Transformers (ViTs) have been shown to outperform CNNs in certain scenarios, particularly when dealing with smaller datasets where CNNs may overfit. The self-attention mechanism inherent in transformers allows for better contextual understanding of the image data, which can enhance classification performance, especially in complex medical imaging tasks [27]. Additionally, studies have indicated that transformers can achieve higher accuracy rates in specific applications, suggesting that they may be more effective in certain diagnostic contexts compared to traditional CNNs [28]. The comparative analysis of CNN and Transformer models in the context of PD diagnosis has been a focal point of recent research. For instance, Sherwani et al. [26] highlighted that Vision Transformers (ViTs) outperform CNNs in scenarios with limited datasets, showcasing their ability to generalize better under such conditions. In contrast, CNNs demonstrate a high level of accuracy rates in larger datasets, as evidenced by studies that reported accuracies exceeding 97% using CNN architectures like VGG16 and ResNet50 [29].

### Key contribution of the proposed article

The key contribution of the proposed studies are as follows:

- The real-time MRI image dataset used in this study was compiled involving sourcing high-quality images, ensuring ethical compliance, preprocessing the data, and organizing it into structured formats suitable for DL analysis.
- A comprehensive comparative analysis was conducted between traditional CNN’s and Transformer-based models for PD classification. This involved implementing multiple CNN architectures and transformers, evaluating their performance on key metrics to benchmark against state-of-the-art (SOTA) approaches.
- A generalizability assessment was performed to validate the robustness of the best-performing model on an independent dataset, Parkinson’s Progression Markers Initiative (PPMI), in order to confirm the model’s capacity in maintain competitive accuracy and stable performance beyond the training domain.
- A quantum-based classification framework was further explored by integrating a Quantum Neural Network (QNN) model to assess its performance against classical deep learning methods, demonstrating the potential of quantum computation for enhancing discriminatory capability and model efficiency in PD classification.
- To improve the interpretability and clinical reliability of the proposed QNN framework, Explainable AI techniques were employed to visualize the regions contributing to the model’s classification decisions.

### Outline of the proposed article

Section “Introduction” (Introduction) highlights the clinical significance of early PD detection and the challenges in conventional diagnostic approaches. Section “Materials and methods” (Materials and Methods) details the dataset used (2.1 Data Collection and Compilation), the CNN architectures implemented (2.2 Conventional CNNs), and the Transformer-based models explored (2.3 Transformer-Based Models). Additionally, 2.4 Model Evaluation and Cross-Validation explain the performance assessment strategies, including K-fold cross-validation. Section “Results” (Results) presents a comparative analysis of model performance, highlighting the effectiveness of CNNs and Transformers in automated feature extraction and classification. Section “Discussion” (Discussion) interprets the findings and discusses on limitations and future directions: Finally, Sect. “Conclusions” (Conclusion) summarizes key contributions, emphasizing AI-driven advancements in PD diagnosis.

## Materials and methods

**Fig. 1:**
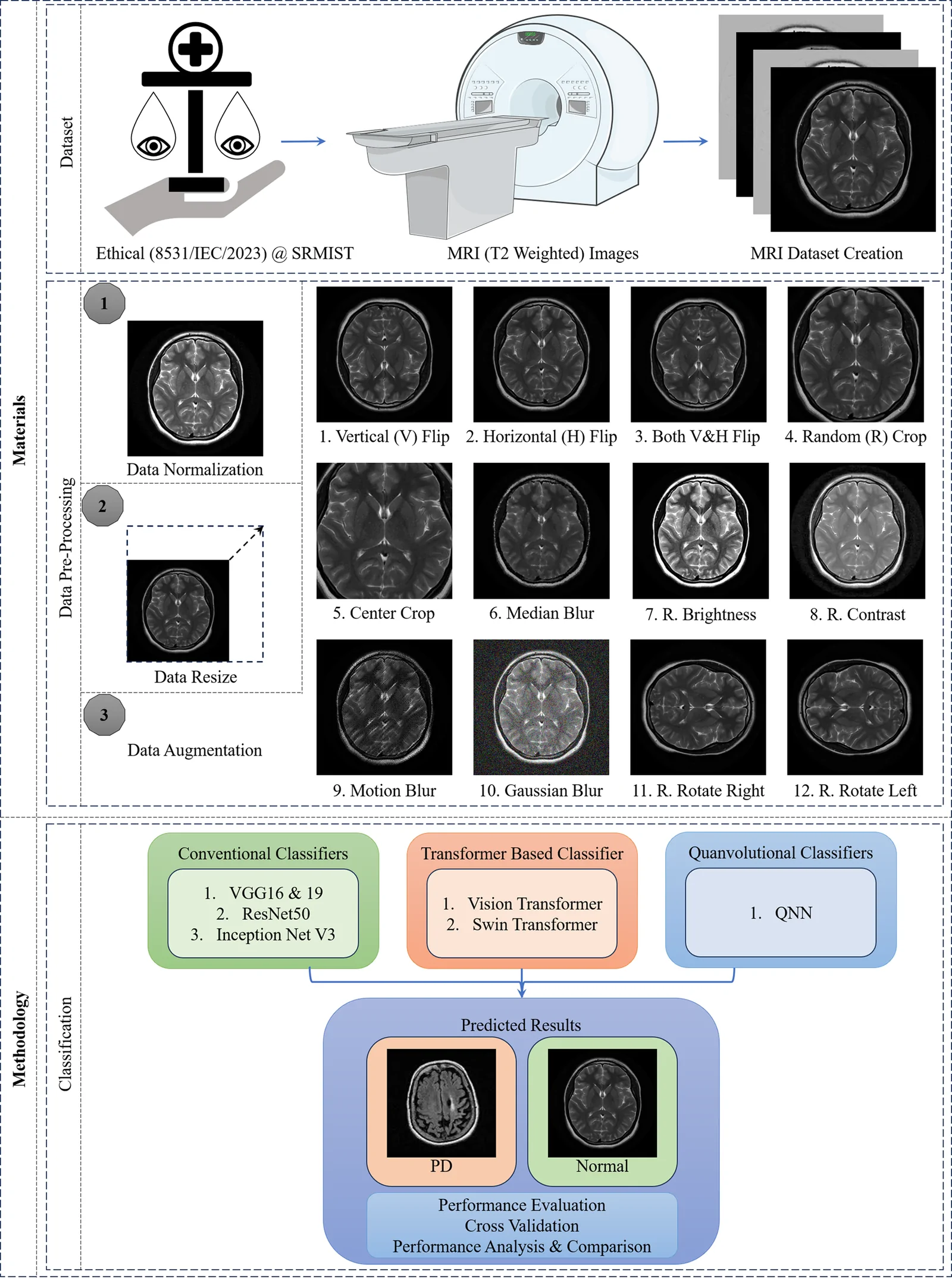
Pipeline of the proposed study

The Fig. 1 illustrates pipeline of the study for MRI-based classification of PD using DL techniques. The process commences with MRI acquisition, where brain scan images are obtained from the subjects followed by multiple preprocessing steps, including normalization, resizing and augmentation.

The preprocessed image dataset serves as input for a various classification framework. Conventional deep learning classifiers such as VGG16 & VGG19 [30], ResNet50 [31], and Inception Net V3 [32] are employed alongside transformer-based models, including Vision Transformer [33] and Swin Transformer [34]. In addition, a QNN-based classification architecture is incorporated to explore the potential of quantum computing in enhancing learning efficiency and decision boundaries for PD classification. The classification framework distinguishes between PD-affected and normal brain scans. The performance evaluation is conducted, followed by comparative analysis to determine the most effective model. This hybrid approach aims to enhance diagnostic accuracy and reliability in the detection of PD from MRI images.

### Data collection and compilation

The T1-weighted MRI primarily provides structural information but lacks the contrast required to detect modest neurodegenerative changes in PD [35]. The T2-weighted MRI image is superior to T1-weighted MRI for PD diagnosis due to its enhanced sensitivity in detecting pathological changes in the substantia nigra, a key region affected in PD [35]. T2-weighted imaging highlights iron accumulation which appears as hypointense regions in the substantia nigra and serves as a biomarker for neurodegeneration [36].

Ethical clearance for this study was secured from SRMIST, Kattankulathur, under the approval number 8531/IEC/2023. Prior to data collection, informed consent was obtained from all the participating volunteers to safeguard the rights and privacy of the individuals involved in the study and to ensure adherence to ethical guidelines. The dataset utilized in this research comprises a total of 300 T2-weighted MRI brain scans, formatted in the Digital Imaging and Communications in Medicine (DICOM) standard. The T2-weighted MRI scans used in this study were acquired at SRM Medical College Hospital and Research Centre using a Siemens clinical MRI scanner operating at a field strength of 1.5 Tesla. The imaging protocol followed standard clinical neuroimaging practices, with a slice thickness of approximately 4 mm and an inter-slice gap ranging from 0 to 20%. The T2-weighted images were obtained using routine clinical settings with standard diagnostic protocols. The dataset includes subjects within the age range of 18 - 60 years, comprising both male and female participants. The inclusion criteria were defined based on clinically relevant cases suitable for PD analysis, while pregnant individuals and paediatric subjects were excluded from the study.

The dataset is evenly distributed, consisting of 150 images from individuals diagnosed with Parkinson’s disease, and 150 images from normal individuals. This balanced dataset facilitates an unbiased analysis, ensuring that the classification models developed in this study are trained and validated on an equal representation of both positive and negative cases. A panel of two domain experts performed the annotation process on the dataset. Each image was examined and classified by both the experts following a consensus-based approach, ensuring high inter-rater reliability. The experts labelled the images into two distinct categories: PD and normal. This manual annotation process aimed to enhance the dataset’s reliability and serve as a gold standard for subsequent model training and validation. In addition to the locally curated dataset used for model training, validation, and testing, the PPMI dataset was employed for independent evaluation to rigorously assess the generalizability of the proposed model across heterogeneous data sources. In this investigation, a subset comprising 113 T2-weighted MRI images from PD patients and 54 images from healthy controls were utilized from the PPMI repository [37, 38].

The implementation of the proposed methodologies, including data preprocessing and classification, was carried out using Google Colab. The cloud-based platform provided an efficient environment for executing DL models and faster computations.

The MRI images were resized and normalized to enhance the efficiency and accuracy of subsequent analysis. Resizing ensures uniformity in image dimensions, which is essential for DL models that require consistent input sizes. This process involves resampling the MRI images to a predefined resolution of 512 × 512. Subsequently normalization was performed to standardize the pixel intensity values across images, thereby improving model convergence and reducing sensitivity to variations in image acquisition settings.

A systematic dataset division was adopted to ensure a rigorous model evaluation, reducing overfitting and improving diagnostic accuracy in distinguishing Parkinson’s disease from normal brain scans. A subset of 50 images from each class were allocated for testing, ensuring an unbiased assessment of model performance. From the remaining 100 images per class, an 80:20 split was performed to obtain training and validation subsets, respectively. Subsequently, extensive data augmentation techniques were applied to the training and validation sets to enhance the diversity and robustness of the model. This resulted in 1,040 images per class for training and 260 images per class for validation. Consequently, the combined dataset across both classes consisted of 2,080 training images and 520 validation images, as summarized in Table 1. This structured partitioning and augmentation facilitates effective model evaluation, enabling performance assessment on unseen data. A detailed distribution of the self-curated dataset used for training, validation, and testing of the model in the classification of PD and normal brain MRI images and PPMI dataset for generalizability assessment is provided in Table 1.

**Table 1:** Data split of the proposed study

Dataset Type	Self-collected Dataset			PPMI Dataset
	Training	Validation	Testing	Generalizability assessment
Parkinson’s Disease	1040	260	50	113
Normal	1040	260	50	54
Total	2080	520	100	167

### Conventional CNNs

#### VGG-16 and VGG-19

**Fig. 2:**
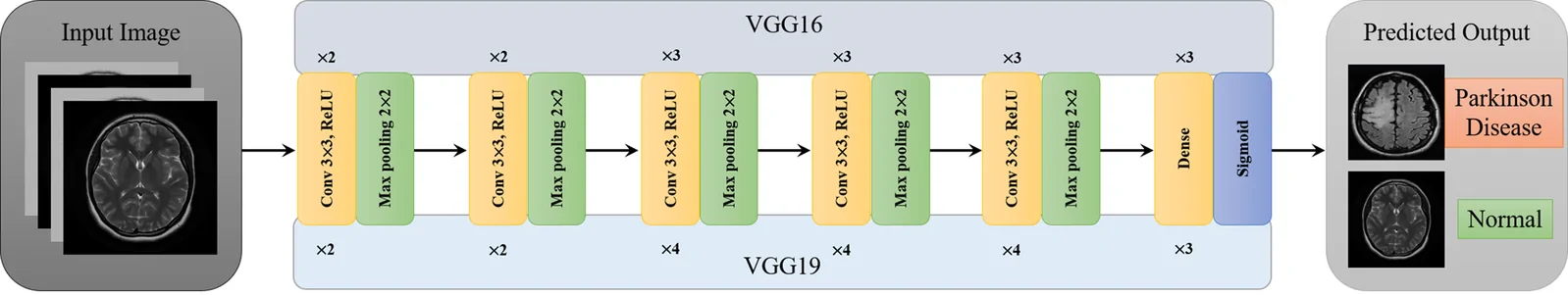
Block diagram of VGG16 and VGG19

VGG16 and VGG19 [30] are deep CNN architectures characterized by a sequential arrangement of convolutional and fully connected layers demonstrating a strong performance in image classification tasks. VGG16 consists of 16 weight layers, including 13 convolutional layers and 3 fully connected layers, while VGG19 extends this with 19 weight layers by adding additional convolutional layers as depicted in Fig. 2. Both architectures employ homogeneous 3 × 3 convolutional filters with a unit stride and ReLU activation to introduce non-linearity, coupled with 2 × 2 max-pooling for spatial down sampling. The classification is achieved via a series of fully connected layers culminating in a SoftMax activation. The deep architecture facilitates effective hierarchical feature extraction, rendering them applicable to domains such as medical image analysis including PD classification. However, the substantial parameter count inherent in these models results in significant computational overhead and memory footprint.

#### ResNet-50

**Fig. 3:**
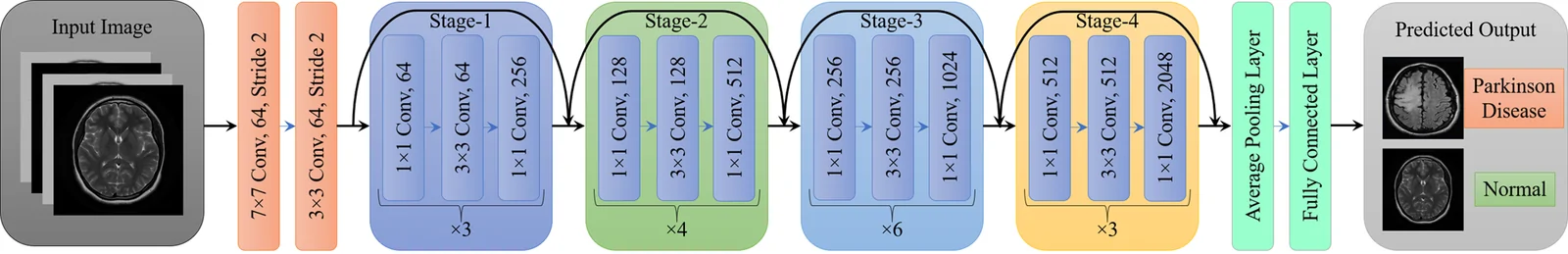
Block diagram of ResNet50

ResNet50 [31], a 50-layer CNN architecture, mitigates the vanishing gradient problem inherent in deep networks by implementing residual learning. This is achieved through the incorporation of skip connections, which bypass multiple layers during backpropagation, thereby facilitating efficient training. The architecture illustrated in Fig. 3 comprises a series of residual blocks, each structured with identity mappings and a bottleneck layer consisting of 1 × 1, 3 × 3, and 1 × 1 convolutional layers, augmented by batch normalization to enhance training stability.

A global average pooling layer precedes the final fully connected layer, effectively reducing overfitting. The residual connections enable ResNet50 to attain high classification accuracy with reduced computational complexity compared to deeper models. Notably, this architecture demonstrates robust performance in medical image classification, owing to its ability to discern complex feature representations, rendering it a suitable solution for deep learning-based diagnostic systems.

#### Inception net V3

**Fig. 4:**
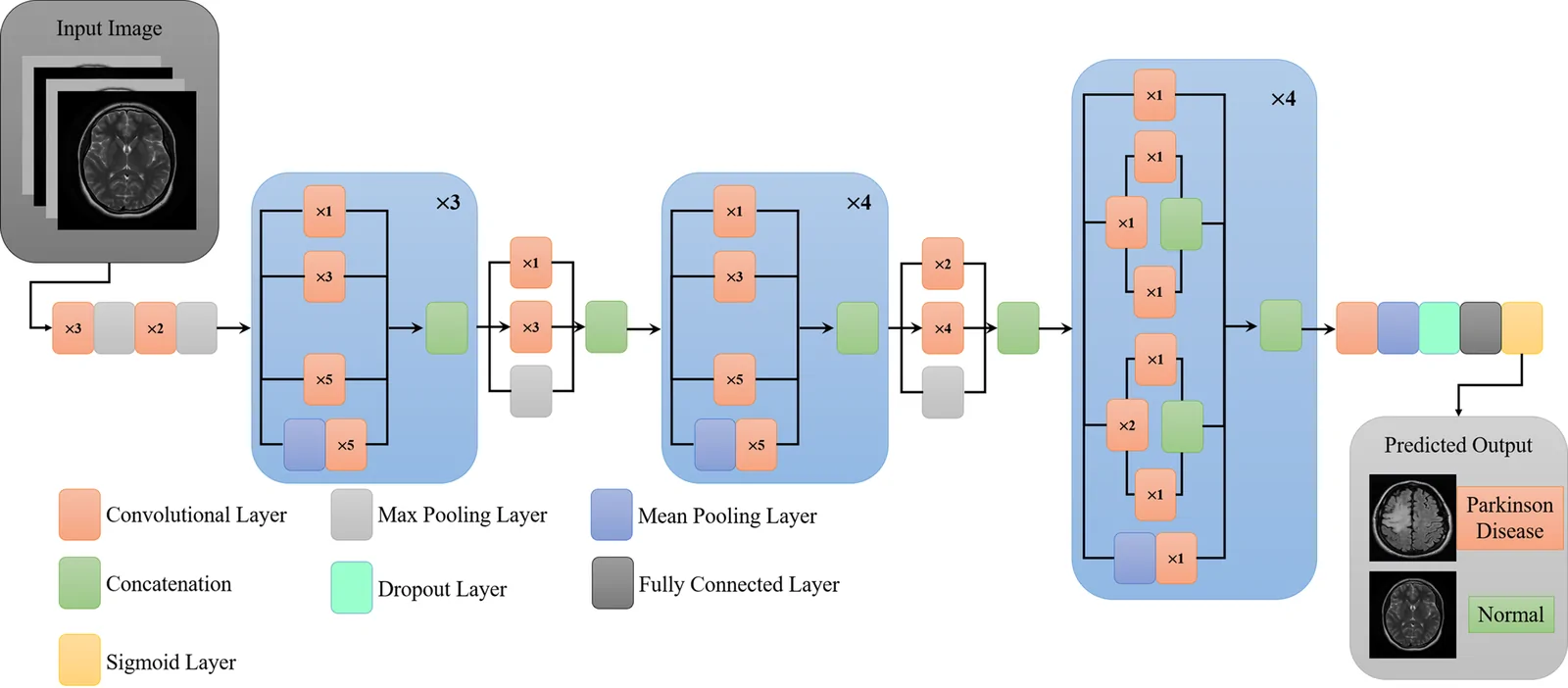
Block diagram of Inception net V3

Inception Net [32], specifically Inception-v3 is designed to enhance feature extraction efficiency by utilizing multi-scale convolutional operations within the same layer. The architecture employs Inception modules as shown in Fig. 4 consisting of parallel convolutional layers with different kernel sizes (1 × 1, 3 × 3, and 5 × 5), along with max-pooling layers, to capture spatial hierarchies at multiple scales. The 1 × 1 convolution plays a crucial role in dimensionality reduction, reducing computational costs while preserving essential features. Inception-v3 has the ability to extract diverse features with reduced redundancy.

### Transformer based models

#### Vision transformers (ViT)

**Fig. 5:**
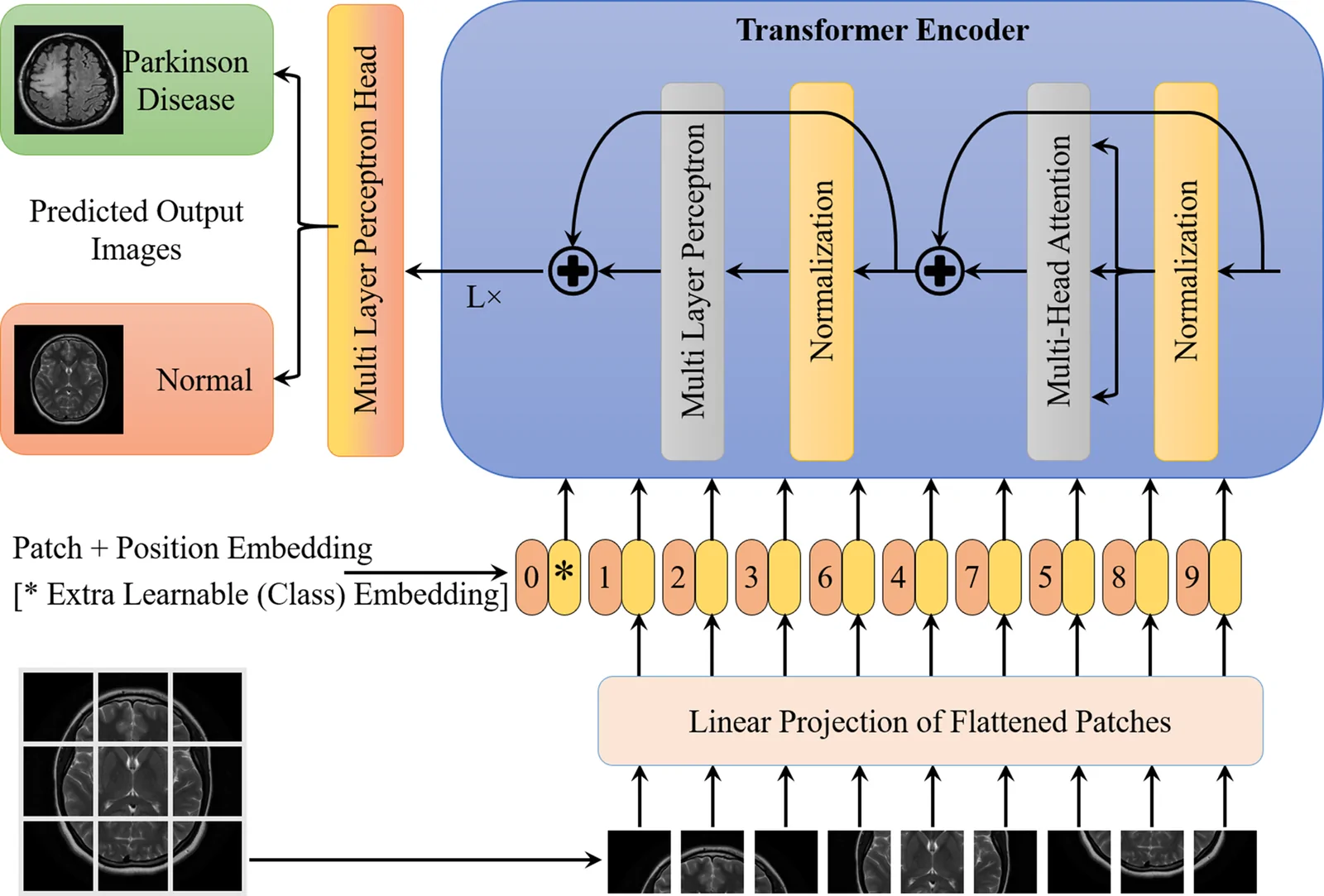
Block diagram of ViT

Unlike conventional CNNs, ViT [33] leverages self-attention mechanisms to process image data in a non-sequential manner, eliminating the need for convolutional operations. Divide an input image into a grid of non-overlapping patches; this is how ViT works, where each patch is treated as a flattened vector as depicted in Fig. 5. These patch embeddings are linearly projected into a latent space using a trainable weight matrix, forming the input tokens for the transformer encoder. To preserve spatial structure, learnable positional encodings are added to the tokenized inputs. The sequence is then fed into multiple self-attention layers, where the model learns complex dependencies interconnected areas inside the picture. One way that ViT is able to successfully capture long-range interactions is through its self-attention mechanism and leverage large-scale pretraining datasets to enhance performance. The final output is derived from a class token, which aggregates global image information and is passed through a classification head. However, the computational cost of ViT is significantly higher than traditional CNNs, necessitating the use of high-performance system.

#### Swin transformers

**Fig. 6:**
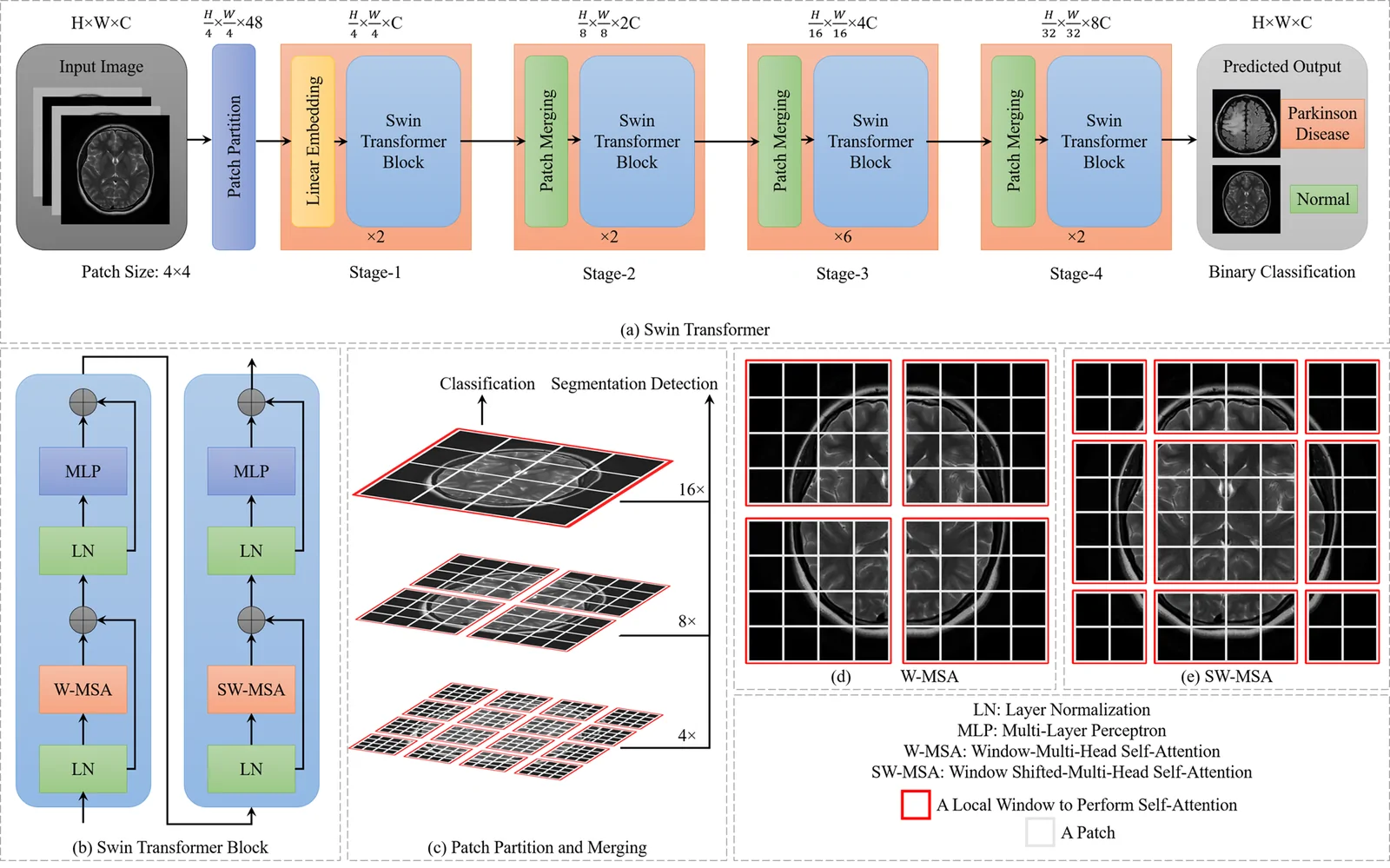
Block diagram of Swin Transformer

The Swin Transformer [34] introduces a hierarchical feature representation and a shifted window mechanism. Swin Transformer constructs a multi-scale feature pyramid, allowing it to capture both fine-grained and high-level semantic information. To ensure cross-window interactions, Swin Transformer employs a shifted window approach in alternating transformer blocks as shown in Fig. 6. In the first stage, self-attention is applied within fixed windows, while in the second stage, window partitions are shifted by half the window size, enabling information flow across neighboring windows. This design significantly reduces the computational complexity compared to standard transformers, where both local texture details and global structures are essential for accurate classification.

### QNN

QNNs represent a computational paradigm that integrates the learning capabilities of artificial neural networks (ANN) with the principles of quantum mechanics. QNNs leverages quantum bits (qubits), existing in superposition and entangled states, enabling a significantly richer representation space for complex learning tasks. This allows the QNNs to process large-scale, high-dimensional data with improved efficiency over traditional DL models. QNN architectures are typically constructed through parameterized quantum circuits (PQCs), where tunable quantum gates encode learnable parameters analogous to weights in classical networks. During training, a quantum-aware optimization algorithm iteratively updates these parameters by minimizing a cost function. The forward pass involves quantum state preparation, application of quantum operations, and measurement of observable values, while the backward pass relies on gradient-based or gradient-free optimization techniques such as parameter-shift rules, genetic optimization, or Bayesian frameworks.

Furthermore, properties such as entanglement using controlled-NOT (CNOT) gates with optimization (parameter-shift rules) allow QNNs to model complex, non-linear correlations that may be difficult to capture using classical neural networks, particularly in fields involving intricate data structures such as medical imaging, genomics, financial forecasting, materials discovery, and cryptography. Hybrid quantum-classical frameworks have gained significant attention, where quantum circuits perform feature transformation or kernel mapping, and classical networks finalize the prediction stage. Current quantum hardware is still considered Noisy Intermediate-Scale Quantum (NISQ) technology, characterized by short coherence times, gate errors, and limited qubit counts, which may degrade learning performance.

**Fig. 7:**
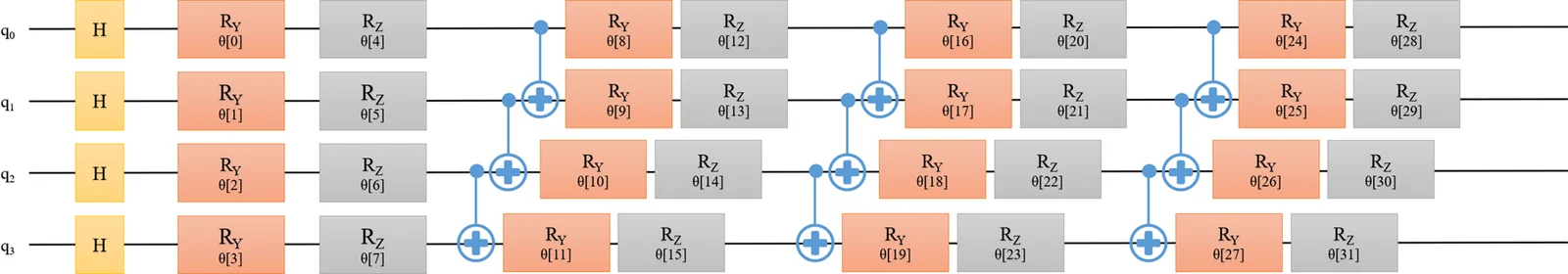
Block diagram of QNN pipeline

Additionally, training QNNs can be computationally expensive due to repeated circuit evaluations and the need for specialized hardware access. Another challenge is the barren plateau phenomenon, where gradients vanish as the quantum system scales, hindering efficient optimization. Ongoing research focuses on designing noise-resilient circuits, improved quantum-aware cost functions, and hardware-efficient ansatz structures. The QNN architecture employed in this investigation using 4 qubits is depicted in Fig. 7. the proposed quantum model was implemented using the Qiskit framework. The PQC was designed with 4 qubits, where classical features were encoded using an angle encoding strategy. The circuit architecture consists of multiple variational layers, each comprising parameterized single-qubit (R_y_(θ) and R_z_(θ)) rotation gates followed by an entanglement block implemented using CNOT gates in a linear entanglement topology. The QNN circuit considered in this study, contains 32 trainable (4 qubits × 2 rotation gates {R_y_(θ) and R_z_(θ)} × 4 layers) parameters. A classical optimizer, specifically the Adam optimizer, was employed to iteratively update the circuit parameters within a hybrid quantum-classical training loop.

Among the CNN variants that demonstrated strong performance, the proposed architecture replaces the conventional convolutional layers with a QNN to enhance feature extraction and classification capability within the deep learning pipeline.

Initially, Hadamard gates were applied to all qubits to generate quantum superposition states prior to feature encoding. The encoded quantum state can be mathematically represented as:

1 $$\left| {\psi (x, \theta)} \right\rangle = U(\theta )U(x){H^{ \otimes n}}{\left| 0 \right\rangle ^{ \otimes n}}$$

where $U\left( x \right)$ denotes the angle encoding operation and $U\left( \theta \right)$ represents the trainable variational quantum circuit composed of parameterized ${R_y}\left( \theta \right)$ and ${R_z}\left( \theta \right)$ gates with linear CNOT entanglement layers. The classical output was obtained using the expectation value of the Pauli-Z observable defined as:

2 $$f\left( x \right) = \left\langle {\psi (x,\theta )\left| {{{\hat Z}^ \wedge }} \right|\psi (x,\theta )} \right\rangle $$

The selection of a 4-qubit architecture was motivated by hardware-efficient circuit design, reduced optimization instability, and computational feasibility within the Qiskit simulation environment.

### Model evaluation and cross validation

All the models were trained using the Adam optimizer, with a batch size of 32 and a dropout rate of 0.5 to mitigate overfitting. The maximum number of training epochs was set to 50; however, an early stopping strategy was employed to prevent unnecessary training once the model performance stabilized. Hyperparameter tuning was performed, with particular emphasis on optimizing the learning rate (0.01, 0.001, 0.0001) to ensure efficient convergence and improved classification performance prior to model evaluation.

The performance metrics of the classifiers were thoroughly evaluated to assess their effectiveness in distinguishing between PD and normal classes [39, 40]. These metrics computed provide a quantitative measure of the classifiers’ predictive capabilities, ensuring a reliable assessment of their diagnostic potential. Additionally, confusion matrices were generated to visualize the distribution of predictions and misclassifications, aiding in understanding the model’s strengths and limitations.

The k-fold cross-validation strategy was employed to check for any potential biases arising from the train-test split [41, 42]. The final performance metrics were then calculated by averaging the results across all *k* iterations. This methodology allowed to assess the model’s performance stability and reduce the risk of overfitting to a particular data partitioning [43, 44].

## Results

Initially, all models were trained using a range of learning rates to determine the optimal value. The performance of each model corresponding to the selected learning rates is summarized in Table 2. Based on this analysis, as the learning rate of 0.0001 yielded the best performance, it was adopted for subsequent validation and testing of the models. A standard Early Stopping strategy was implemented during training by monitoring the validation loss (val_loss) metric to prevent overfitting and unnecessary training iterations. Early stopping was achieved when no significant improvement in accuracy or loss was observed for three consecutive epochs.

**Table 2:** Accuracy achieved by each CNN and Transformer-based architecture during hyperparameter tuning across different learning rates

Architecture	Learning Rate = 0.01		Learning Rate = 0.001		Learning Rate = 0.0001	
	Epoch	Accuracy	Epoch	Accuracy	Epoch	Accuracy
VGG16	50/50	0.7942	50/50	0.8144	42/50	0.8798
VGG19	50/50	0.7995	50/50	0.8394	39/50	0.8904
ResNet50	50/50	0.8240	50/50	0.8659	29/50	0.9322
InceptionNetV3	50/50	0.8168	50/50	0.8481	36/50	0.9202
ViT	50/50	0.8466	50/50	0.8928	12/50	0.9688
Swin	50/50	0.8875	50/50	0.9221	10/50	0.9712

The performance evaluation of traditional DL architectures, transformers and QNN considered in this investigation, conducted based on multiple metrics across both training and validation phases are presented in Tables 3, 4 and 5 respectively. The comparative analysis of CNNs and transformer-based architectures reveals that transformer-based models exhibit superior performance across all key evaluation metrics. The Swin Transformer achieved the accuracy of 0.9712 during training and 0.9673 during validation among the transformer models, followed closely by ViT. Among the CNN models, the accuracy demonstrates a progressive improvement, with ResNet50 achieving the highest values of 0.9322 in training and 0.9192 in validation, followed closely by Inception Net V3. Fall-out and miss-rate, which represent false positive and false negative rates, respectively, show lower values for Swin Transformer, highlighting its reliability. Precision, NPV, FDR, and FOR further reinforce this trend, with Swin Transformer consistently outperforming other traditional models. Overall, while CNN-based architecture, ResNet50 deliver strong performance, transformer-based models, particularly the Swin Transformer, demonstrate superior generalization, higher classification accuracy, and reduced false detection rates. The QNN achieved a highest training accuracy of 0.9851 and validation accuracy of 0.9731, compared to CNN and transformer-based models, reflecting excellent generalization with minimal overfitting.

**Table 3:** Evaluation of traditional deep learning architectures performance

Metrics	Training				Validation			
Architecture	VGG16	VGG19	ResNet50	Inception Net V3	VGG16	VGG19	ResNet50	Inception Net V3
Accuracy	0.8798	0.8904	0.9322	0.9202	0.8692	0.8827	0.9192	0.9096
Sensitivity	0.8827	0.8933	0.9365	0.9240	0.8846	0.9000	0.9385	0.9269
Specificity	0.8769	0.8875	0.9279	0.9163	0.8538	0.8654	0.9000	0.8923
Fall-Out	0.1231	0.1125	0.0721	0.0837	0.1462	0.1346	0.1000	0.1077
Miss-Rate	0.1173	0.1067	0.0635	0.0760	0.1154	0.1000	0.0615	0.0731
Precision	0.8776	0.8881	0.9285	0.9170	0.8582	0.8699	0.9037	0.8959
NPV	0.8820	0.8926	0.9360	0.9234	0.8810	0.8964	0.9360	0.9243
FDR	0.1224	0.1119	0.0715	0.0830	0.1418	0.1301	0.0963	0.1041
FOR	0.1180	0.1074	0.0640	0.0766	0.1190	0.1036	0.0640	0.0757
F1-Score	0.8802	0.8907	0.9325	0.9205	0.8712	0.8847	0.9208	0.9112

**Table 4:** Evaluation of vision and Swin transformer architectures performance

Metrics	Training		Validation	
Architecture	ViT	Swin	ViT	Swin
Accuracy	0.9688	0.9712	0.9558	0.9673
Sensitivity	0.9721	0.9750	0.9654	0.9769
Specificity	0.9654	0.9673	0.9462	0.9577
Fall-Out	0.0346	0.0327	0.0538	0.0423
Miss-Rate	0.0279	0.0250	0.0346	0.0231
Precision	0.9656	0.9676	0.9472	0.9585
NPV	0.9719	0.9748	0.9647	0.9765
FDR	0.0344	0.0324	0.0528	0.0415
FOR	0.0281	0.0252	0.0353	0.0235
F1-Score	0.9689	0.9713	0.9562	0.9676

**Table 5:** Evaluation of proposed QNN architecture performance

Metrics	Training	Validation
Accuracy	0.9851	0.9731
Sensitivity	0.9837	0.9654
Specificity	0.9865	0.9808
Fall-Out	0.0135	0.0192
Miss-Rate	0.0163	0.0346
Precision	0.9865	0.9805
NPV	0.9837	0.9659
FDR	0.0135	0.0195
FOR	0.0163	0.0341
F1-Score	0.9851	0.9730

As the QNN and Transformer-based models have demonstrated superior performance than the DL models examined in this study, the data partitioning for the QNN and Transformer models was validated using K-fold cross-validation. The outcomes obtained by the ViT, Swin Transformer and QNN are detailed for each fold, along with the mean and standard deviation (SD) across all folds in Tables 6, 7 and 8, respectively.

**Table 6:** K-fold cross-validation results of vision transformer architecture performance

Metrics	Fold-1	Fold-2	Fold-3	Fold-4	Fold-5	Mean ± SD
Accuracy	0.9481	0.9635	0.9519	0.9558	0.9596	0.9558 ± 0.0054
Sensitivity	0.9538	0.9769	0.9577	0.9615	0.9654	0.9631 ± 0.0079
Specificity	0.9423	0.9500	0.9462	0.9500	0.9538	0.9485 ± 0.0039
Fall-Out	0.0577	0.0500	0.0538	0.0500	0.0462	0.0515 ± 0.0039
Miss-Rate	0.0462	0.0231	0.0423	0.0385	0.0346	0.0369 ± 0.0079
Precision	0.9430	0.9513	0.9468	0.9506	0.9544	0.9492 ± 0.0039
NPV	0.9533	0.9763	0.9572	0.9611	0.9650	0.9626 ± 0.0079
FDR	0.0570	0.0487	0.0532	0.0494	0.0456	0.0508 ± 0.0039
FOR	0.0467	0.0237	0.0428	0.0389	0.0350	0.0374 ± 0.0079
F1-Score	0.9484	0.9639	0.9522	0.9560	0.9598	0.9561 ± 0.0055

**Table 7:** K-fold cross-validation results of Swin transformer architecture performance

Metrics	Fold-1	Fold-2	Fold-3	Fold-4	Fold-5	Mean ± SD
Accuracy	0.9692	0.9596	0.9712	0.9654	0.9635	0.9658 ± 0.0041
Sensitivity	0.9808	0.9692	0.9769	0.9731	0.9654	0.9731 ± 0.0054
Specificity	0.9577	0.9500	0.9654	0.9577	0.9615	0.9585 ± 0.0051
Fall-Out	0.0423	0.0500	0.0346	0.0423	0.0385	0.0415 ± 0.0051
Miss-Rate	0.0192	0.0308	0.0231	0.0269	0.0346	0.0269 ± 0.0054
Precision	0.9586	0.9509	0.9658	0.9583	0.9617	0.9591 ± 0.0049
NPV	0.9803	0.9686	0.9767	0.9727	0.9653	0.9727 ± 0.0054
FDR	0.0414	0.0491	0.0342	0.0417	0.0383	0.0409 ± 0.0049
FOR	0.0197	0.0314	0.0233	0.0273	0.0347	0.0273 ± 0.0054
F1-Score	0.9696	0.9600	0.9713	0.9656	0.9635	0.9660 ± 0.0041

**Table 8:** K-fold cross-validation results of proposed QNN architecture performance

Metrics	Fold-1	Fold-2	Fold-3	Fold-4	Fold-5	Mean ± SD
Accuracy	0.9692	0.9731	0.9769	0.9750	0.9712	0.9731 ± 0.0030
Sensitivity	0.9766	0.9805	0.9844	0.9654	0.9615	0.9737 ± 0.0098
Specificity	0.9621	0.9659	0.9697	0.9846	0.9808	0.9726 ± 0.0097
Fall-Out	0.0379	0.0341	0.0303	0.0154	0.0192	0.0274 ± 0.0097
Miss-Rate	0.0234	0.0195	0.0156	0.0346	0.0385	0.0263 ± 0.0098
Precision	0.9615	0.9654	0.9692	0.9843	0.9804	0.9722 ± 0.0098
NPV	0.9769	0.9808	0.9846	0.9660	0.9623	0.9741 ± 0.0096
FDR	0.0385	0.0346	0.0308	0.0157	0.0196	0.0278 ± 0.0098
FOR	0.0231	0.0192	0.0154	0.0340	0.0377	0.0259 ± 0.0096
F1-Score	0.9690	0.9729	0.9767	0.9748	0.9709	0.9729 ± 0.0031

To investigate the possibility of data leakage introduced by data augmentation, an additional 5-fold cross validation experiment was performed where initially, 160 (80 PD and 80 Normal) original MRI images were augmented using 13 different augmentation techniques, resulting in 2,080 (1040 PD and 1040 Normal) augmented training images. An independent validation set consisting of 40 (20 PD and 20 Normal) original MRI images was retained without applying any augmentation, thereby ensuring that the validation samples remained completely unseen during training. The augmented training images (2,080) and the original validation images (40) constituted a total dataset of 2,120 images. Subsequently, a 5-fold cross-validation strategy was adopted using an 80:20 split. This experiment was solely performed to ensure that this fold-wise separation preserved the independence of the validation data throughout the evaluation process. The proposed validation protocol was applied consistently across the ViT, Swin Transformer, and QNN models and the results are presented in Tables 9, 10 and 11, respectively.

**Table 9:** 5-fold cross-validation performance of ViT model under data leakage assessment

Metrics	Fold-1	Fold-2	Fold-3	Fold-4	Fold-5	Mean ± SD
Accuracy	0.9528	0.9623	0.9552	0.9505	0.9575	0.9557 ± 0.0046
Sensitivity	0.9401	0.9717	0.9406	0.9361	0.9575	0.9492 ± 0.0142
Specificity	0.9662	0.9712	0.9683	0.9659	0.9575	0.9658 ± 0.0053
Fall-Out	0.0338	0.0288	0.0317	0.0341	0.0425	0.0342 ± 0.0053
Miss-Rate	0.0599	0.0283	0.0594	0.0639	0.0425	0.0508 ± 0.0142
Precision	0.9670	0.9717	0.9406	0.9361	0.9575	0.9546 ± 0.0151
NPV	0.9387	0.9528	0.9707	0.934	0.9575	0.9507 ± 0.0140
FDR	0.0330	0.0283	0.0594	0.0639	0.0425	0.0454 ± 0.0151
FOR	0.0613	0.0472	0.0293	0.0660	0.0425	0.0493 ± 0.0140
F1-Score	0.9535	0.9717	0.9406	0.9361	0.9575	0.9519 ± 0.0139

**Table 10:** 5-fold cross-validation performance of SwinT model under data leakage assessment

Metrics	Fold-1	Fold-2	Fold-3	Fold-4	Fold-5	Mean ± SD
Accuracy	0.9693	0.9741	0.9693	0.967	0.9623	0.9684 ± 0.0038
Sensitivity	0.9585	0.9631	0.9585	0.9583	0.9495	0.9576 ± 0.0049
Specificity	0.9806	0.9855	0.9807	0.9760	0.9757	0.9797 ± 0.0042
Fall-Out	0.0194	0.0145	0.0193	0.0240	0.0243	0.0203 ± 0.0042
Miss-Rate	0.0415	0.0369	0.0415	0.0417	0.0505	0.0424 ± 0.0049
Precision	0.9811	0.9858	0.9811	0.9764	0.9764	0.9802 ± 0.0040
NPV	0.9575	0.9623	0.9575	0.9575	0.9481	0.9566 ± 0.0051
FDR	0.0189	0.0142	0.0189	0.0236	0.0236	0.0198 ± 0.0040
FOR	0.0425	0.0377	0.0425	0.0425	0.0519	0.0434 ± 0.0051
F1-Score	0.9693	0.9741	0.9693	0.9670	0.9623	0.9684 ± 0.0038

**Table 11:** 5-fold cross-validation performance of QNN model under data leakage assessment

Metrics	Fold-1	Fold-2	Fold-3	Fold-4	Fold-5	Mean ± SD
Accuracy	0.9811	0.9741	0.9764	0.967	0.9835	0.9764 ± 0.0063
Sensitivity	0.9722	0.9631	0.9676	0.963	0.9767	0.9685 ± 0.0054
Specificity	0.9904	0.9855	0.9856	0.9806	0.9905	0.9865 ± 0.0042
Fall-Out	0.0096	0.0145	0.0144	0.0194	0.0095	0.0135 ± 0.0042
Miss-Rate	0.0278	0.0369	0.0324	0.0370	0.0233	0.0315 ± 0.0054
Precision	0.9906	0.9858	0.9858	0.9811	0.9906	0.9868 ± 0.0039
NPV	0.9717	0.9623	0.9671	0.9619	0.9765	0.9679 ± 0.0056
FDR	0.0094	0.0142	0.0142	0.0189	0.0094	0.0132 ± 0.0039
FOR	0.0283	0.0377	0.0329	0.0381	0.0235	0.0321 ± 0.0056
F1-Score	0.9813	0.9744	0.9766	0.9720	0.9836	0.9776 ± 0.0047

The independent testing dataset used for final model evaluation was completely isolated prior to augmentation and was never involved in the training or validation process. Therefore, the primary performance metrics reported for the proposed framework remain unaffected. Furthermore, the inclusion of an external independent PPMI dataset for generalization assessment provides additional evidence regarding the stability and applicability of the proposed framework beyond the self-curated dataset.

**Fig. 8:**
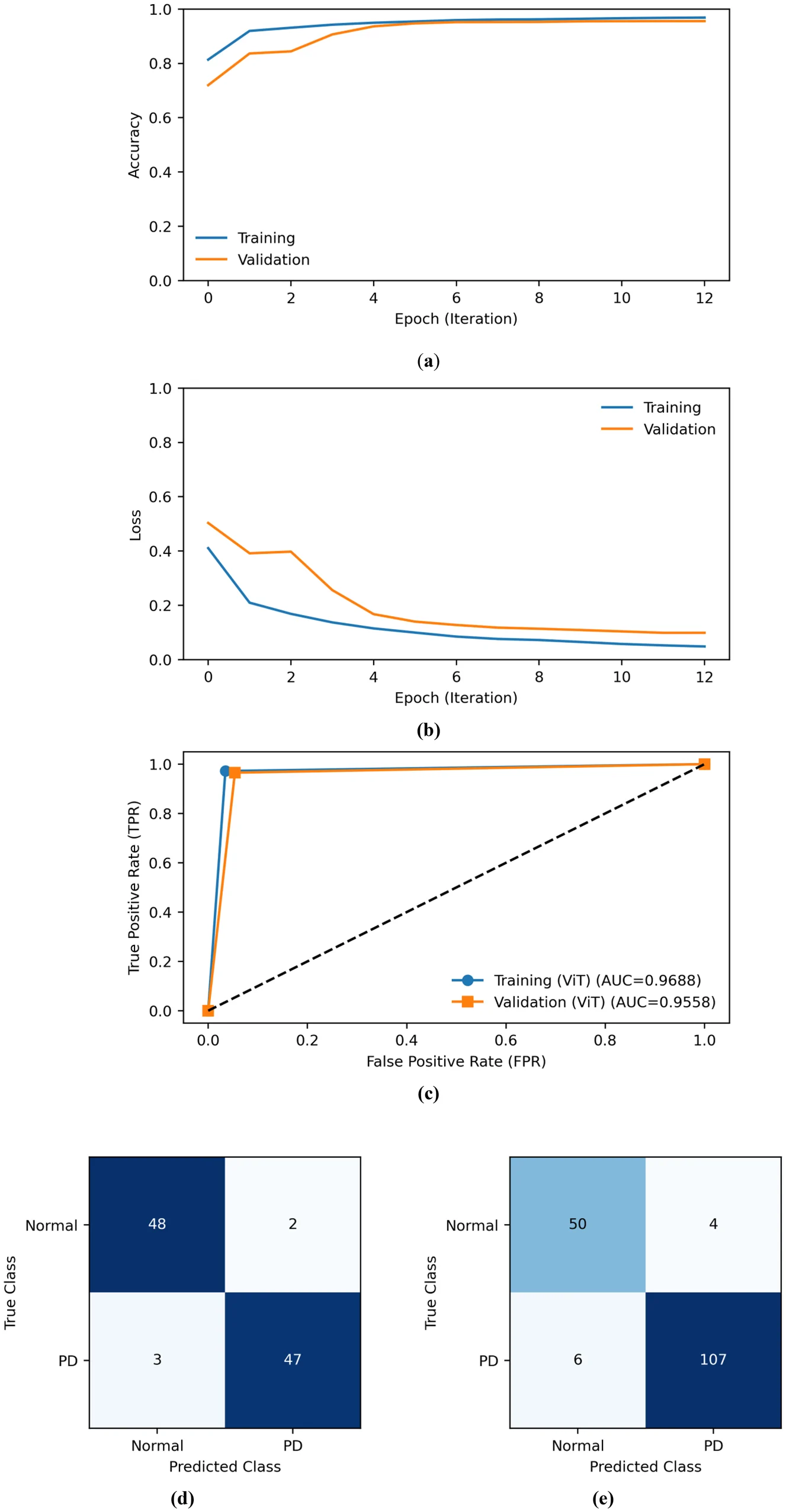
Performance evaluation plots of the vision Transformer (ViT): (**a**) Accuracy curve, (**b**) Loss curve, (**c**) Receiver operating characteristic (ROC) curve (**d**) Confusion matrix-testing results and (**e**) Confusion matrix—generalizability assessment results

**Fig. 9:**
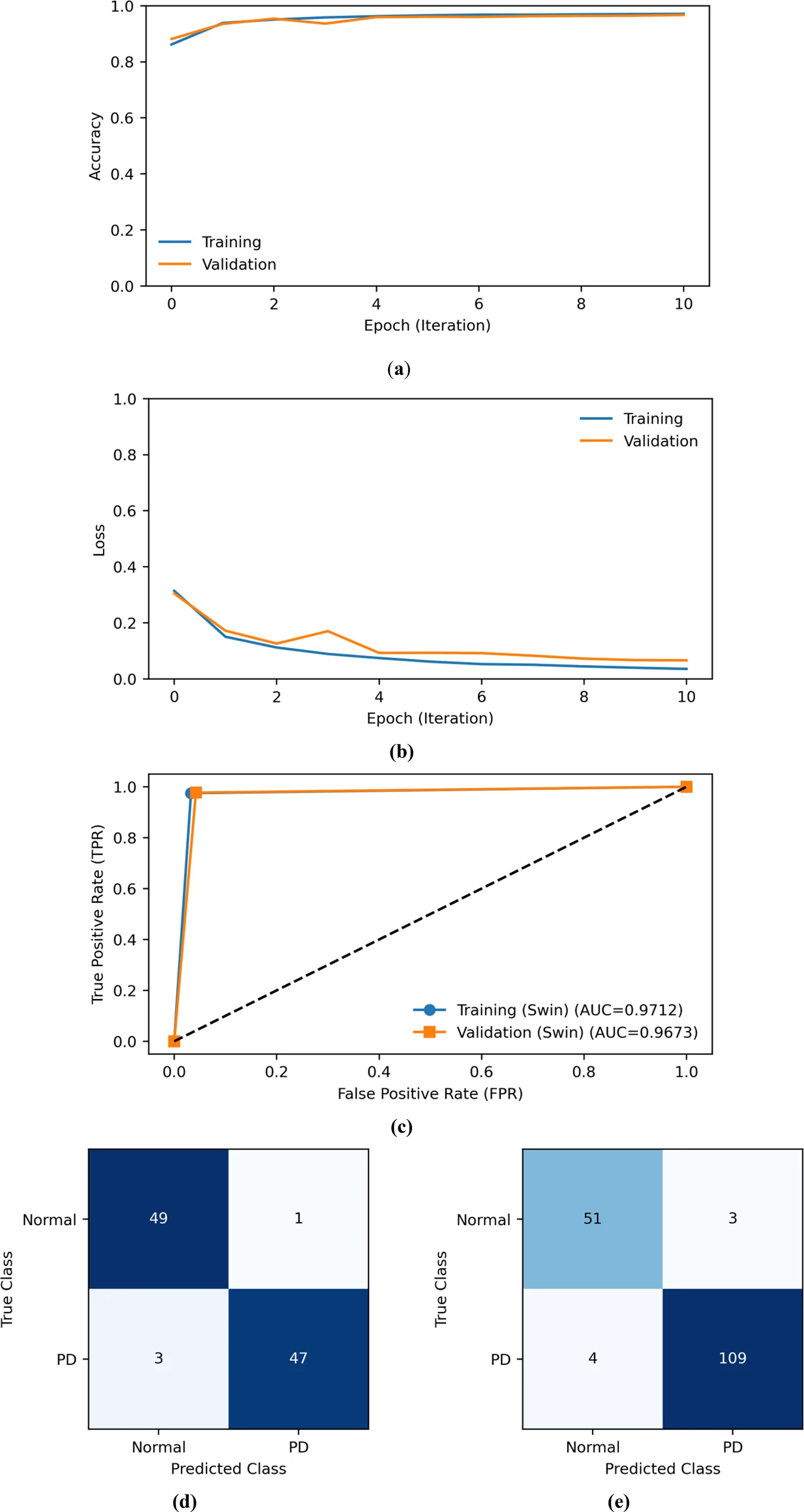
Performance evaluation plots of the Swin Transformer: (**a**) Accuracy curve, (**b**) Loss curve, (**c**) Receiver operating characteristic (ROC) curve (**d**) Confusion matrix-testing results and (**e**) Confusion matrix—generalizability assessment results

**Fig. 10:**
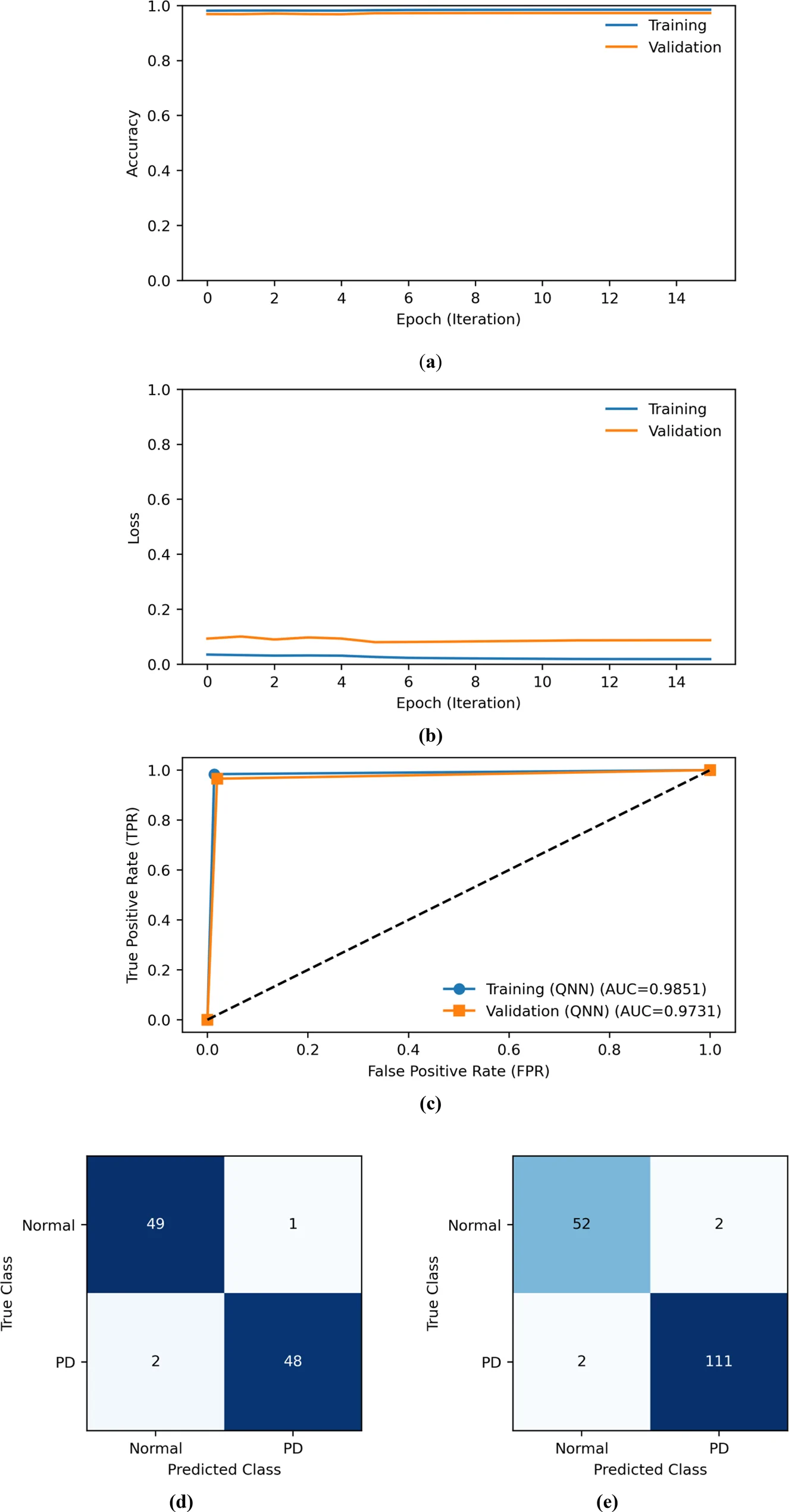
Performance evaluation plots of the proposed QNN: (**a**) Accuracy curve, (**b**) Loss curve, (**c**) Receiver operating characteristic (ROC) curve (**d**) Confusion matrix-testing results and (**e**) Confusion matrix—generalizability assessment results

As the accuracy of the QNN and transformer models are superior than other DL models considered in this study, the model accuracy and loss curves of ViT, Swin Transformer and QNN for both training and validation datasets over multiple epochs are presented in Figs. 8 (a & b), 9 (a & b) and 10 (a & b).

The Receiver Operating Characteristic (ROC) curve for the ViT, Swin Transformer and QNN, illustrating the model’s ability to differentiate between categories is presented in Figs. 8 (c), 9 (c) and 10 (c). The confusion matrix of ViT, Swin Transformer and QNN on the test images, depicted in Figs. 8 (d), 9 (d) and 10 (d) indicates low misclassification rates. The confusion matrices corresponding to the ViT, Swin Transformer and QNN models during the generalizability assessment are presented in Figs. 8 (e), 9 (e) and 10 (e) respectively.

**Fig. 11:**
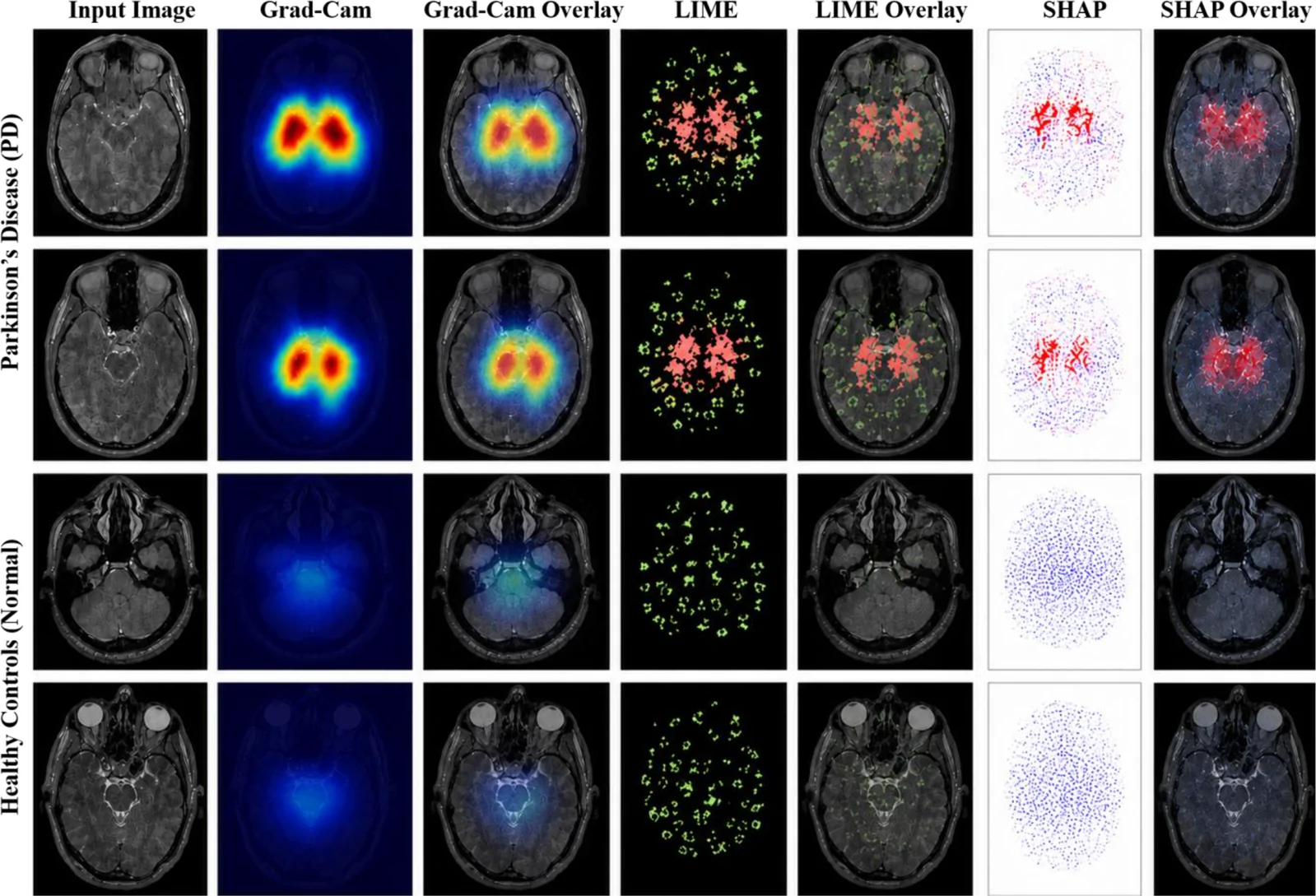
Explainable AI-based visualization of PD and normal brain images

To improve the interpretability and clinical reliability of the proposed QNN framework, Explainable AI techniques were employed to visualize the regions contributing to the model’s classification decisions. Figure 11 presents representative visual explanations for both PD and Normal brain MRI images. The figure demonstrates the original MRI image, the heatmaps, local and global feature attribution visualizations and their corresponding overlay on the original image.

The Gradient-weighted Class Activation Mapping (Grad-CAM) heatmaps reveal high-intensity activations (red and yellow regions), indicating that the proposed model primarily focuses on clinically relevant brain regions while making diagnostic decisions. The corresponding overlay images further confirm that the highlighted discriminative regions coincide with neuroanatomical structures known to be affected by PD pathology.

The local explanation maps identified using Local Interpretable Model-agnostic Explanations (LIME) highlights the image super pixels that contribute positively and negatively to the final prediction. Regions represented in red indicate positive evidence supporting the PD classification, whereas green regions highlight less influential features. The feature attribution maps generated using SHapley Additive exPlanations (SHAP) quantify the contribution of individual image features to the model’s prediction. Red regions represent features with positive contributions that increase the confidence of the PD prediction, whereas blue regions indicate features with negative contributions that reduce the prediction confidence.

## Discussion

The evaluation of CNN architectures presented in Table 2 for PD detection demonstrates that ResNet50 achieves the most robust classification performance, excelling in accuracy while maintaining the lowest error rates. ResNet50 closely follows Inception Net V3 and outperforms VGG19 and VGG16 in all assessed metrics, reflecting its enhanced capability in distinguishing features relevant to PD detection. The notably lower fall-out and miss-rate values of ResNet50 highlight its ability to minimize false detections. While all architectures display commendable classification efficiency, ResNet50 and Inception Net V3 stand out as the most proficient models, delivering superior predictive accuracy in PD diagnosis.

The comparative assessment of ViT and Swin Transformer architectures indicates that both models exhibit high classification performance, with Swin Transformer slightly outperforming ViT across all metrics. While both ViT and Swin Transformer demonstrate strong classification efficiency than the CNN models, Transformer emerges as the more optimal architecture, offering enhanced performance and robustness for medical image analysis. The comparative analysis of the performance characteristics of both 80:20 split and K-fold suggests that both the Transformer models are not overly sensitive to specific data splits implying a better degree of generalizability.

**Fig. 12:**
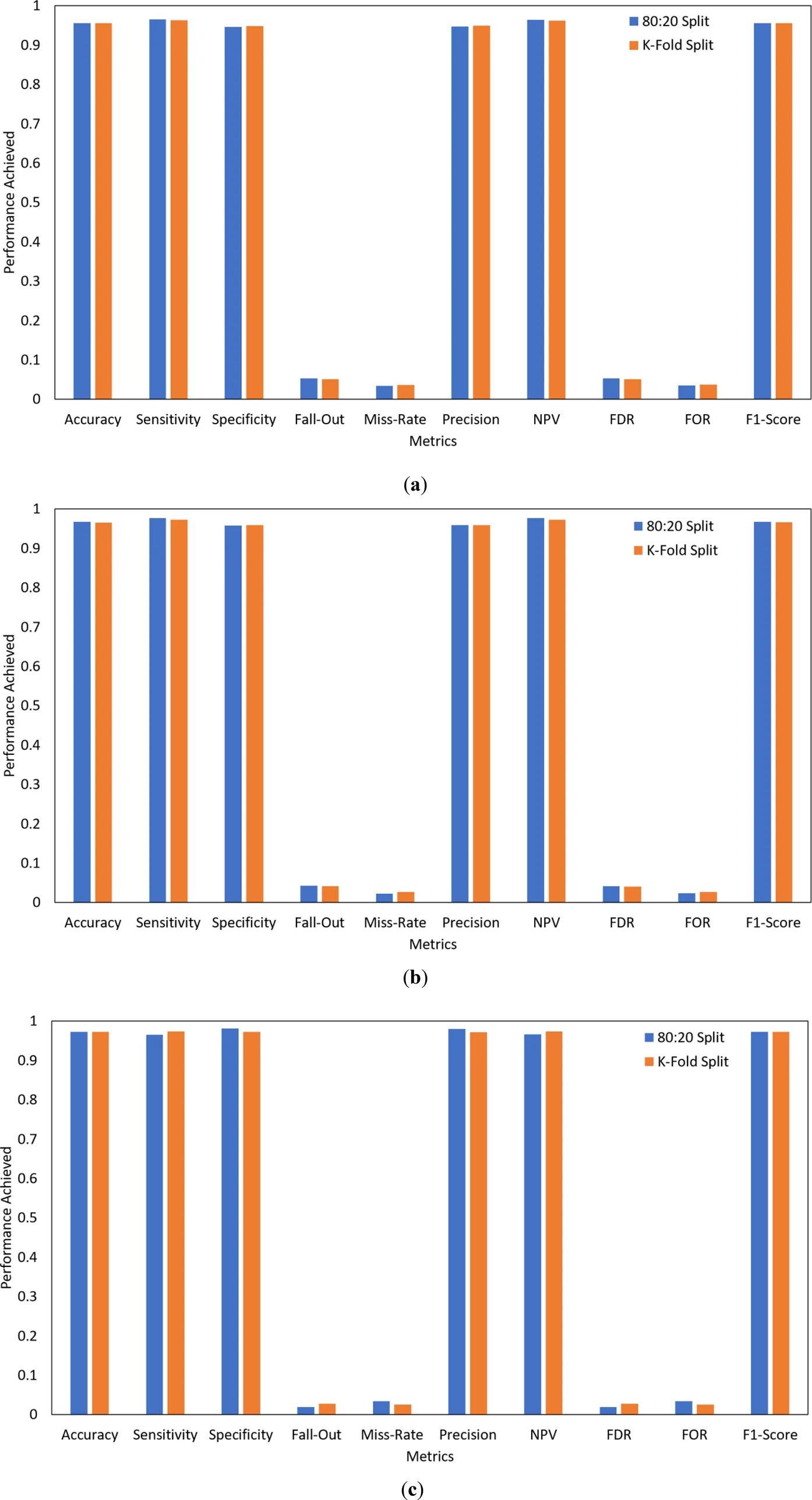

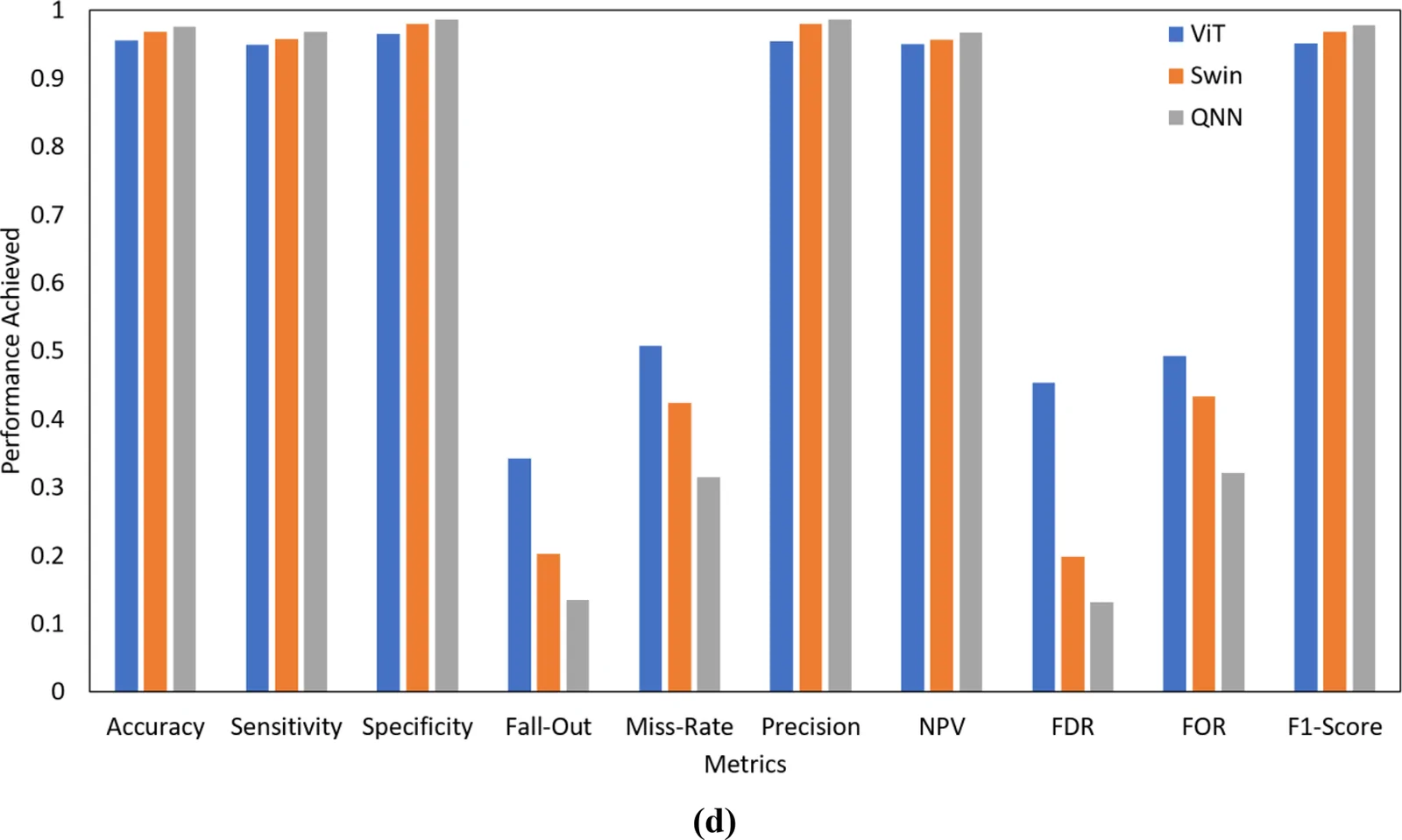
Comparative analysis of data splits: (**a**) ViT split evaluation, (**b**) Swin transformer split evaluation, (**c**) QNN split evaluation and (**d**) Comparison of classification performance under a data leakage assessment experiment

A comparative bar chart, depicted in Fig. 12 (a-c) delineates the performance of the ViT, Swin Transformer and QNN models under two distinct evaluation paradigms: a single 80:20 train/test split and a K-fold cross-validation procedure. The close concordance between the performance metrics obtained through these two methods indicates a lack of significant variance attributable to data partitioning.

The classification performance of the 5-fold cross validation carried out data leakage assessment is illustrated in Fig. 12 (d). It was observed that all three models exhibited comparable performance under the leakage-free evaluation strategy, demonstrating that the reported classification results are robust.

In the accuracy curve, initially there is a rapid increase, while the loss curves show a rapid decrease, indicating effective learning during the early epochs. As training progresses, the accuracy and loss stabilize, demonstrating convergence towards optimal performance. The close alignment of training and validation accuracy and loss curves suggests minimal overfitting, indicating that the model generalizes well to unseen data. This performance evaluation confirms the effectiveness of the Transformer framework in classifying MRI images with high accuracy.

The near-perfect ROC curves, suggest that the model achieves excellent sensitivity and specificity. The Area Under the Curve (AUC) values for training and validation indicates a high discriminative capability of the model. The minimal gap between training and validation AUC values further indicates that the model generalizes well to unseen data, demonstrating its effectiveness in MRI-based classification tasks.

The confusion matrix illustrating the performance of the QNN, demonstrates high accuracy with minimal misclassifications compared to ViT and Swin Transformer models. The ViT correctly identifies 48 positive cases and 47 negative cases, while only misclassifying 2 negative cases as positive and 3 positive cases as negative. Swin Transformer successfully classifies 49 instances as positive and 47 as negative, while making 1 incorrect positive prediction and 3 incorrect negative predictions. the QNN correctly identified 48 Parkinson’s cases and 49 normal cases, highlighting its accurate discriminative learning. Only 1 normal subject was misclassified as PD, while 2 PD cases were incorrectly labeled as normal.

The confusion matrix for the ViT during the generalizability assessment indicates that out of 113 PD cases, 107 were correctly identified, with only 6 misclassified as normal controls. Similarly, among 54 normal images, 50 were accurately recognized, while 4 were incorrectly classified as PD. These results correspond to an overall accuracy of 94.6% maintaining a competitive accuracy compared to the accuracy attained with testing dataset. The Swin Transformer during the generalizability assessment achieved 95.8% accuracy with 160 correct classifications and 7 misclassifications for both PD and normal conditions. The confusion matrix results further validate the effectiveness of the QNN framework. As illustrated, the model correctly classified 111 PD cases (True Positives) and 52 normal subjects (True Negatives), demonstrating strong capability in learning discriminative MRI-based features. Misclassification rates were minimal, with only 2 normal cases incorrectly predicted as PD (False Positives) and 2 PD labeled as normal (False Negatives). This balanced distribution of prediction errors emphasizes the stability and reliability of the QNN model across both classes. The low false-positive and false-negative rates indicate that the QNN not only minimizes unnecessary clinical alarms but also substantially reduces the risk of missed detections. Overall, the results confirm the high diagnostic confidence of the QNN model, supporting its applicability for computer-aided PD screening.

### Limitations and future directions

The promising performance of DL in PD diagnosis using MRI scans, this study has certain limitations. Firstly, the dataset used is limited in size and may not fully represent the diverse population affected by PD, potentially impacting model generalizability. Secondly, while CNN and Transformer-based architectures were evaluated, other hybrid or multi-modal deep learning approaches were not explored, which could further enhance classification accuracy. In addition, the Quantum Neural Network (QNN) model, despite demonstrating reliable classification capability, is constrained by current limitations in quantum hardware and simulation environments. The QNN experiments were conducted on classical simulators due to the restricted availability and scalability of contemporary quantum processors, which may not fully reflect real quantum execution performance. Moreover, noise, qubit decoherence, and limited qubit counts remain significant barriers that restrict complex model architectures and deeper circuit designs. Future research can address these limitations by expanding the dataset and exploring multi-modal DL approaches. For QNN-based research, future work will include benchmarking on real quantum hardware, developing noise-resilient quantum circuits, and exploring quantum-classical hybrid learning schemes to leverage the strengths of both computational paradigms. The investigation will be further extended to multi-class models including the other sub-classes such as Multiple System Atrophy (MSP) and Progressive Supranuclear Palsy (PSP).

## Conclusions

This research highlights the effectiveness of QNN and DL techniques in automating PD diagnosis in MRI images. By leveraging CNN, Transformer-based and QNN models, their ability to distinguish PD-affected brains from healthy controls was assessed. The QNN demonstrated the highest classification performance, while Swin Transformer and ResNet50 emerged as the most effective Transformer and CNN-based model. The stability of the results, confirmed through K-fold cross-validation, reinforces the reliability of 80:20 approach. Importantly, a generalizability assessment using the independent dataset further confirms that the best-performing models sustained high accuracy when exposed to heterogeneous external data. The findings of this investigation emphasize the transformative impact of AI in medical imaging, offering an efficient and objective diagnostic tool for PD detection. The DL framework holds significant potential for integration into clinical workflows, aiding neurologists in timely and accurate diagnosis. Future research should focus on expanding datasets and improving model interpretability to enhance trust and adoption in clinical practice.

## Data Availability

The data that support the findings of this study are privately available at the following URL https://www.ppmi-info.org/access-data-specimens/download-data (accessed on 06 August 2026); https://doi.org/10.1002/acn3.644.
